# From “cracking the orthographic code” to “playing with language”: toward a usage-based foundation of the reading process

**DOI:** 10.3389/fpsyg.2014.00891

**Published:** 2014-08-22

**Authors:** Sebastian Wallot

**Affiliations:** Department of Culture and Society and Interacting Minds Centre, Aarhus UniversityAarhus, Denmark

**Keywords:** reading research, natural reading, meaning, language use, language games

## Abstract

The empirical study of reading dates back more than 125 years. But despite this long tradition, the scientific understanding of reading has made rather heterogeneous progress: many factors that influence the process of text reading have been uncovered, but theoretical explanations remain fragmented; no general theory pulls together the diverse findings. A handful of scholars have noted that properties thought to be at the core of the reading process do not actually generalize across different languages or from situations single-word reading to connected text reading. Such observations cast doubt on many of the traditional conceptions about reading. In this article, I suggest that the observed heterogeneity in the research is due to misguided conceptions about the reading process. Particularly problematic are the unrefined notions about meaning which undergird many reading theories: most psychological theories of reading implicitly assume a kind of elemental token semantics, where words serve as stable units of meaning in a text. This conception of meaning creates major conceptual problems. As an alternative, I argue that reading shoud be rather understood as a form of language use, which circumvents many of the conceptual problems and connects reading to a wider range of linguistic communication. Finally, drawing from Wittgenstein, the concept of “language games” is outlined as an approach to language use that can be operationalized scientifically to provide a new foundation for reading research.

## LANGUAGE USE AND READING – WHY BOTHER?

Reading is a “culturally cognitive” phenomenon that sets humans apart from other intelligent creatures. Theoretically, reading is interesting because it is a learned practice that incorporates many human capacities; from basic processes of visual perception to abstract cognitive skills such as reasoning, imagination, and creativity. The ability to read and comprehend texts has become a key necessity for participation in contemporary society: it is a prerequisite for all forms of higher education (Rindermann and Ceci, 2009), and has direct consequences for health and life expectancy (Pignone and DeWalt, 2006). Accordingly, the empirical investigation of the reading process has one of the longest traditions in experimental psychology, dating back more than 125 years.

However, despite this long tradition, the scientific understanding of reading has made rather heterogeneous progress: much progress has been made in uncovering many facts about reading, highlighting how linguistic, individual and situational factors influence the process of reading under certain circumstances. However, this progress in gathering facts about reading and its constituent factors has not been complemented by a similar theoretical progress that pulls together the observed facts. This is reflected in the complex patterns of contextual effects on reading behavior that pervade the scientific literature (Van Orden et al., 2001) and a rather fragmented theoretical landscape (Rayner and Reichle, 2010).

Moreover, and in addition to the problems inherited from strict experimental investigations, recent findings on connected text reading, literary reading, and cross-language investigations of reading have started to gnaw at the edges of accepted assumptions, as they seem to indicate that some of the major theoretical commitments in most of reading research – such as the primacy of the word level, the importance of lexical features, or the assumption that words have a definite meaning – do not apply to naturalistic, or at least more complex reading situations.

In this essay, I explore the possibility that the observed heterogeneity and non-convergence in reading research is due to a somewhat misguided conception of the reading process: up to now, reading research has been very much concerned with the front-end of the reading process (i.e., how visual features can be correctly identified as words) and the search for general mechanisms that are invariant across contexts (Pollatsek et al., 2003). Conceptually, reading is seen as a rather passive process. Other fields that concern themselves with how language works, such as the philosophy of language or interaction and communication research, have largely moved on from a strict mechanistic view to a usage-based view of language. In a nutshell, this means that the function of linguistic tokens have no life of their own, but are first and foremost subject to temporal and contextual factors, turning the theoretical priorities of contemporary reading research on its head.

In what follows, I will discuss the possibility that the theoretical priorities in psychological reading research have to be reconsidered. Especially, that some of the theoretical core-commitments of contemporary reading research might be incommensurable with necessary assumptions about reading as a language phenomenon. To explain my reasoning, I will first give a brief description of what would seem to be indispensable ingredients of reading as a phenomenon that would probably be agreed upon by the vast majority of psychologists, philosophers and scholars of literature. Then, I will summarize the core-commitments of contemporary research on reading and evaluate how these commitments relate to the minimal core-assumptions one has to make for reading as a language phenomenon. Based on this review, I will conclude that the empirical results and fundamental concerns about meaning are somewhat at odds with the current core-assumptions of reading research. And furthermore that the conceptual problems that arise in this context might be solved by adopting a more usage-based view on reading, where reading is conceptualized less as a translational process that “maps printed words into the mind,” but more as a “one-and-a-half-person dialog” of sorts. I will finish by picking up Wittgenstein’s concept of “language games” and describe how this concept can be used as a linchpin for a general understanding of communicative processes, linking reading with online communication, and how this allows the deduction of the concept of “reading games”, that can potentially serve as a new core-foundation for reading research in empirical investigation of the reading process.

## WHAT IS READING FOR?

Reading and writing are relatively recent cultural developments: the earliest records of the precursors of writing data back to cave paintings roughly 20,000 years old, and the first traces of proto-writing appeared 3,400 BC in the Middle East, the Proto-Cuneiform. This pictographic form seems to have been first invented for bookkeeping purposes. In an interesting way similar to rather modern developments of emailing and texting, these communications were of a rather short lived nature: as Nissen (1993) notes “After authorized individuals have broken sealed stoppers of collars in order to gain access (…), the fragmented sealings may have been kept somewhere for control purposes but then lost their purpose and were consequently disposed of. Written documents were unquestionably treated in the same way. They served to carry out future check (…). After a certain time had lapsed, this information was no longer useful. Concequently, the tables were probably thrown away in regular intervals (…) (p. 6).” Hence, early instances of reading and writing were much more connected to their environment, serving rather direct, sign-post like functions.

It took at about another 600 years for the first appearance of coherent texts that would qualify as literature (Grimbly, 2013), and yet roughly another 1000 years until the first alphabetic languages appeared (Sampson, 1985). These developments in the conventionalization of writing systems finally served to establish writings as a more permanent medium for communication across broader scales, where authors could present their thoughts to an increasingly bigger audience. Finally, printing techniques allowed for increasingly efficient multiplication and simultaneous distribution to several places at once – broadening the space for communication.

Through writing, authors could preserve their thoughts, allowing communication on new temporal scales, even past the lifespan of an author. Besides this one-author-one-reader relationship, reading of texts also tied in with online communication between groups of people, as many individuals could now read the same text and discuss, interpret, and act upon its content (for example, the concept of the newspaper, or the air-dropping of pamphlets during war time). Of course, modern informational technology has also created a space in-between those two – the classical reading situation and the classical form of online communication – where emails, short messages, and chats allow a more fast-paced, tightly coupled exchange that uses reading and writing as means to transport content within the setting of online communication. Hence, it seems generally acceptable to say “Reading (and writing) is a form of communication, and it evolved to serve a communicative function.”

Furthermore, we can refine this statement, by specifying more how reading (and writing) goes about serving that communicative function in a basic way, which is by providing a specific medium, a visual symbol system/writing system that transports content. This is a general aspect that reading shares with all other forms of communication, that transport content by means of some medium – for example, the sound of the voice during reading aloud (but also visual aspects, such as gestures, facial expressions, etc.). Hence, we can say: “Reading is an activity necessary for “accessing” content from the communicative medium of the writing system – necessary in order to “use” a writing system as a communicative medium.”

In the end, one could also say that for something to be labeled a successful communication – or indeed communication at all as opposed to mere activity (and similar to the distinction between action and behavior) – “meaning” needs to be present, or that an activity needs to be meaningful.

What has been said so far might seem trivial, or commonly agreeable upon, but we will see that the details of how these aspects of reading are understood in particular will make quite a difference. We will need some space to unpack this second statement – relating the process of reading to meaning – because it will turn out to be more complicated and in the end maybe more disagreeable than it seems at first glance. It will also mark the first departure of how core-assumptions in psychological reading research are understood or implemented compared to other fields, such as interaction and communication research. The highlighted terms “assess” and “use” stand for different ideas of how one can think about how texts work and will ultimately relate to some notion of “meaning”. In the next section, I will review how ideas of “access”, “content” and “meaning” are related in contemporary reading research, and how they seem to be understood and practically implemented in that field.

## PSYCHOLOGICAL RESEARCH ON READING – “CRACKING THE ORTHOGRAPHIC CODE”

How has reading been conceived in psychology? The earliest systematic investigations of reading probably come from the work of Cattell (1886a,b), who investigated reading on the letter, word, and sentence level using tachistoscopic methods. His research revealed some basic facts about reading that have stood the test of time fairly well, for example that readers can read longer letter strings when these are grouped into real words, as opposed to being random concatenations of letters, and that the latency in sounding out a monosyllabic word is shorter compared to sounding out a single letter. Based on these findings, his conclusion was that reading was a synthetic process, in which a word was read and recognized by a reader as a whole.

These and their own findings prompted Erdmann and Dodge (1898) to formulate a “total shape” theory of reading, describing skilled reading as holistic recognition of words. In particular, they presented evidence that skilled readers that are familiar with a specific vocabulary can identify words as long as 22-letters reliably within very short exposure times of 100 ms (the experiments were conducted in German, where it is possible to compound several words, especially nouns, into a single word). As Scheerer (1981) points out, this prompted one of the first great theoretical debates about the reading process, because Wundt (who had – up to that point – not been particularly interested in empirical reading research), doubted that this was possible. In particular, Wundt (1900) thought that the effective presentation time of words in the tachistoscope was prolonged by after-image effects, and that multiple shifts of attention must have occurred on sub-strings of these extremely long words in order to successfully read them.

The crux of this debate was, whether reading is basically an analytic process (where local details of a word need to be visually analyzed first in order to successfully read it), or a synthetic process (where the word is read as a whole), and this debate came to dominate the theoretical discourse of reading researchers well into the 1960s (Gibson and Levin, 1975).

Another line of reading research that started at the turn of the century was that of eye-movements during reading (Huey, 1908). Early investigations of eye-movements did not directly address the issue of analytic versus synthetic reading, as it was clear that the visual span around foveal vision during a fixation could easily provide information about longer words, even if they were only fixated once. Hence, the shifts of attention that Wundt proposed were unlikely to reveal themselves as a pattern of multiple fixations within a single word. In any case, this research seemed to corroborate the notion of reading as a discrete, word-by-word identification process, whereby individual words are fixated in sequence, and the duration of a fixation was indicative of the skill of a reader (see Quantz, 1897, for the first investigations of skilled reading using the eye-voice span).

Eventually, the debate about word reading as an analytic versus synthetic process was tried to be settled by the introduction of dual-route models (Coltheart, 1978), which incorporated both processes into a single theory of word reading. The basic idea was the reading of a word could either go through a direct route, where the “total shape” of the word was being directly mapped to its representation in the mental lexicon (synthetic reading), or it could go through an indirect route, were the individual letters of the word needed to be recognized and the phonology of that word was reconstructed though its spelling and could be used to map the word to its representation in the mental lexicon (analytic reading). Furthermore, the dual route models also incorporated reading speed as a fundamental variable, as it was hypothesized that the direct route would permit faster word reading compared to the indirect route.

Direct access was assumed to be faster, but contingent on the reader’s familiarity with the word read (Doctor and Coltheart, 1980). This familiarity effect could be captured by the frequency with which a word appears in a language, as a stand-in for the average memory strength evoked by that word for the average reader. Accordingly, word frequency became a central variable that was important for all well-developed reading models, either as an explanatory principle or as a fact to be explained, no matter their specific architecture (Grainger and Jacobs, 1996; Coltheart et al., 2001; Pollatsek et al., 2003; Engbert et al., 2005). Many more lexical variables that described word properties have been subsequently described in an attempt to find the set of relevant lexical word properties that would allow a reader to “crack the orthographic code” and map the visual features of a word to its internal representation.

In general, theories of reading in psychology have been concerned with this “front end” of the reading process, and tried to describe invariant relationships that would permit a reader to map a word to the mental lexicon. Implicitly or explicitly, it seems as if comprehension of a word has been loosely equated with the success of the mapping of word and representation, where the meaning of the word is stored. This way meaning is explicitly tied to the level of words. The (semantic) content of a word equals its meaning. Of course, the number of definitions of meaning of a word are many, but in the end, meaning is treated as a stable word property, at best locally and incrementally developing and only subject to peripheral context effects. When meaning is accepted to be given, then it makes sense to focus on the processes that lead up to it – hence, the focus on “access” (in a broad sense) of the content of a word – or in other words, the focus on how readers crack the orthographic code of a piece of written language. Under the hand, this perhaps implicit view of meaning in reading is one of a word semantics, where the stable, well-definable building blocks of meaning reside on the word level, and any higher forms of meaning can – one way or the other – be reduced to that level. Meaning of any complexity can be decomposed into its elements, its words.

This is not only reflected in the architecture of theories and models of reading, but also in the experimental procedures that are utilized in reading research: here, the focus on isolated words and sentences was deemed sufficient, as they seemed to encapsulate the essentials of written language comprehension: words contain the basic meanings and lexical constituents of written language, while the syntactical features of a language can be sufficiently tabulated within a single sentence (Wallot and Van Orden, 2011). Following this logic, the investigation of isolated words and sentences was thought to contain the potential to uncover the general rules of written language perception.

Just as a little hint at this point: the idea of “language-use” as it is conceptualized in contemporary philosophy and interaction studies does not easily fit into this picture. If anything, it seems that the most plausible interpretation of the term language use in the contemporary view of reading research would be, that it captures the dynamics of the reading process, which in turn would be (entirely) defined by the sequence of the properties of its word-constituents.

However, thinking about the concept of “language use” in reading at this point would be premature, as we have not yet answered the question of why this should be interesting or even relevant. In order to do so, we will concern ourselves with the following two questions in the following two sections: (1) How did the outlined research program in reading research fare so far with its focus on the word level? (2) Given that meaning is a central and ultimately necessary ingredient for reading as a phenomenon, are these assumptions of psychological models and theories in line with plausible definitions of meaning that have been much more pondered upon in the philosophy of language?

## STATE-OF-THE-ART: DO WORD PROPERTIES REVEAL A FUNDAMENTAL LEVEL OF READING?

The experimental investigation of reading has been heavily focusing on stimuli sets of no more than a few words, with studies that are explicitly aimed at more naturalistic text reading encompassing a handful of sentences at the most (Clifton and Duffy, 2001). And indeed, some basic features of the reading process (such as the fixation-saccade-sequence in reading) suggested that reading is inherently word reading. The process of text reading appears very complex, and many reading researchers feel that quite a reduction of complexity is necessary before systematic investigations of reading are possible. Interestingly, Wundt (1911) also argued that an experimental analysis of complex intellectual functions such as reading demanded such a reduction, but he argued that an experimental investigation of the linguistic processes involved in reading might escape such an experimental analysis, and rather needed non-experimental methodologies (Scheerer, 1981).

Contemporary reading researchers are also aware of this tension. For example, Rayner and Pollatsek (1994) state that “Critics of the information-processing approach often argue that attempts to isolate component processes of reading result in tasks very much unlike reading. (…) Admittedly, [many of] these tasks are unlike reading (p. 8).” However, either due to lack of attractive alternatives or as the expression of an optimistic attitude toward scientific progress, the current sentiment still seems to be: “Suppose we are interested in studying walking. If we study the motor responses that people make when they take two steps, critics may say, “But that’s not walking. When you walk you go a long way.” True, but are the motor responses any different when you take two steps? Undoubtely not” (again Rayner and Pollatsek, 1994, p. 8).

Whether one agrees or not, there has certainly been good reason for this linguistic sparcity in reading research, because the basic lexical and syntactical features already show complicated interaction effects with each other. For example, a reaction time recorded by a key-press to read the word “pepper” is on average faster if the word “pepper” is preceded by a semantically related word, such as “salt,” compared to a control condition where “pepper” is preceded by an unrelated word (Neely et al., 1998). However, if “salt” is presented twice in succession, just before “pepper” appears, this facilitative effect vanishes.

All simple reading tasks reveal such complicated patterns of interactions among the factors that are studied in reading research (Van Orden et al., 2001; Pickering et al., 2013), and these interactions are not just limited to the scale of word reading: while, for example, Kintsch and Keenan (1973) found that reading times increase linearly with the syntactic complexity of a sentence, more recent research by Keller et al. (2001) found that this effect is actually dependent on the lexical features of the constituent words of a sentence (i.e., word frequency).

This cursory example might not appear troublesome on its own. However, they are no exceptions to the rule, but are symptomatic for the current state-of-affairs in reading research: Van Orden and Kloos (2005) provide an in-depth discussion of the complicated interaction effects observed in single-word reading research on the role of phonology in reading (see also Van Orden et al., 2001). Intuitively, phonology seems a potentially important aspect of reading, because the development of reading usually follows the development of speech, and because the majority of writing systems incorporate some form of phonological coding into their orthography. Accordingly, as has been described above, phonology is assumed to be an important mediator in the reading process, for example described in the indirect route in dual-process theories (Coltheart, 1978).

In their review, Van Orden and Kloos (2005) argue that effects of phonology in the reading process are fundamentally contingent on the task demands within a specific reading task that is employed to investigate reading, and that this “task condingent evidence, instead of settling the debate [about the role of phonology in reading] simply fuels it (p. 63).”

One example area that Van Orden and Kloos (2005) discuss and that illustrates the pervasiveness of complex interaction effects in reading is the case of homophone errors: homophones are words that sound like another word when spoken, but differ in spelling, which creates conflicting responses from readers – for example in a categorization task, when the words “break” versus “brake” should be categorized as “part of a car.” Skilled readers make homophone errors irrespective of their familiarity, which seemed to falsify the dual process theory where highly familiar words should be recognized via the direct access route that does not incorporate mediating phonology, and it could be concluded that phonology does not matter. However, when the breadth of the category in a categorization task is changed, then readers make more homophone errors for unfamiliar compared to familiar words (Jared and Seidenberg, 1991) indicating that phonology does matter differently for both routes. Whether homophone effects appear in a reading task also depends on other task aspects, such as the general difficulty of the task (Lukatela and Turvey, 1994) or reader’s skill (Unsworth and Pexman, 2003).

As Van Orden and Kloos (2005) conclude, the problem is that reading reveals itself as an ultra-sensitive phenomenon where different aspects of tasks and readers depend on each other. Furthermore, the addition of further factors will rather complicate matters: as the number of factors that are incorporated into the design of reading tasks increases, so does the number of interactions among those factors. Hence, the exquisitely complicated relations between the different factors in reading can never be stably pinned down (at least so far it has not been). Paradoxically, the attempt to identify and isolate the mechanisms that serve as building blocks of the reading process rather gives rise to the conclusion that given circumstance, everything matters. Without being able to pinpoint reliably what matters when, one effectively confronts a situation where “everything is dependent on everything else.”

Regarding the case of phonology in reading, this means that the question cannot be settled. Ideally, laboratory tasks would reveal a robust role for phonology throughout, but given that they don’t, the question has not found a satisfactory answer and creates circumstances in which scientist are not so much informed by the evidence, but rather have to choose which evidence should be given priority (Van Orden et al., 2001). Similar problems have also appeared in the study of reading disorders, where dyslexic readers were found to deviate from normal readers in innumerable aspects of cognitive measures, such as basic perceptual ability, working memory, attention, or temporal processing, but at the same time, none of these measures by themselves provide a sufficient criterion for the diagnosis of dyslexia (Wijnants et al., 2012). This persistent non-convergence of findings from laboratory research on reading has been noticed long since (Van Orden et al., 2003), and reflected in critical assertions that a general theory of reading is nowhere in sight despite a more than 100-year research effort (Rayner, 1998; Rayner and Reichle, 2010).

However, it is important to point out clearly one more time that this state of affairs is already observed in carefully controlled experimental laboratory tasks of reading, and no research program was ever conducted to systematically investigate whether these tasks capture a process that is anywhere akin to naturalistic reading. One of the challenges for reading research in the near future will be to weed-out which of the tasks that were used in laboratory settings to study reading actually generalize to more naturalistic reading situations – and which ones are really only confined to a laboratory life of their own (Hunt and Vipond, 1991; McNerney et al., 2011; Wallot et al., 2013). Three interesting aspects of reading have come to the light of day that seem to question whether reading research has placed its bets on good assumptions: (1) The role of idiosyncrasies in reading, (2) the generalization of research findings across different languages, and (3) the generalization of results of laboratory research to more naturalistic text reading situations.

Idiosyncrasies in reading behavior are long-since known in psychology (Rayner, 1998). What is understood to be an idiosyncratic process does not even lie in the realm of individual interpretations of a text or the like. Rather, what is meant are systematic quantitative or qualitative differences in measures of the reading process between individuals, such as which particular passages in a text evokes emotional responses during reading, how readers move their eyes across a text (for example with few, long fixations versus many short fixations during reading), how often or not they re-read passages of a text, or simply how they differ in reading speed (Miall and Kuiken, 1994; Rayner and Pollatsek, 1994). Even the latter, simple measure can reveal astonishing differences. For example, in a study of text reading of mine (Wallot et al., 2013), participants read simple fictional prose. None of the participants knew the text beforehand, and all were college students, literate native readers in the language the text was presented in. All participants had to answer a comprehension questionnaire after reading. On average, reading of the text took at about one hour. However, it took the slowest reader almost 2.5 h to read the text, while the fastest reader went through it in a little more than 17 min. Yet, both of them were perfectly able to answer the administered comprehension questionnaire (i.e., answering all questions correctly). However, current theories of reading are inherently theories of the average reader. Neither quantitative (such as reading speed), nor qualitative (such eye-movement patterns) idiosyncratic reader differences find any deep consideration in the well-developed theories and models of reading (Grainger and Jacobs, 1996; Coltheart et al., 2001; Pollatsek et al., 2003; Engbert et al., 2005). Moreover, these idiosyncrasies can usually only be observed in somewhat naturalistic or at least complex reading tasks that boast at least a little bit of degrees of freedom for the reader, such as connected text with an overarching or emergent meaning (Hunt and Vipond, 1991; Sikora et al., 2011). However, reading tasks – like most of the tasks utilized in experimental psychology – are explicitly designed to minimize idiosyncratic behavior as much as possible, and in so far as idiosyncrasies make for differences in the reading process or between reading outcomes, they show up as an error term in the experimental study of reading. Current research does not seem to have the conceptual tools to deal with strong idiosyncratic processes in reading, because it requires that the reading process is fundamentally the same across people in the details of how it works, and individual differences are often seen as obstacles that are in the way of such a kind of understanding (Lupker et al., 1997).

While the case of idiosyncratic reading behavior hints at the limits of the current framework that searches for context-free mechanisms, this framework has been put more directly to the test in cross-linguistic investigations of reading: a recent debate has sparked around reading universals, that is, around the aspects of the reading process that are invariant across languages and reading situations (Frost, 2012). After all, if a well-definable, context-independent cognitive architecture supports reading as a cognitive activity, then these building blocks should be the same no matter the language. This also follows from our general consideration, that reading has evolved as a means for human communication via written texts, and that no matter the specific details of a writing system, all these systems are powerful enough to express the same ideas and ideas of the same degree of complexity. It has turned out that what were thought to be basic building blocks of the reading process, such as letter position invariance within a word, do not occur the same way across languages. For example, new research showed that the reading process is relatively robust to the scrambling of the letter positions in a word, pointing to a fundamental property of the mental (and neurophysiological) processes during reading (Frost, 2012). However, it was subsequently shown that this is mostly a phenomenon of European languages, but does not pertain to other languages such as Hebrew, where letter position is of great importance (Velan and Frost, 2007; Velan et al., 2013) – and we want, for now, to cast aside the question of what a concept such as letter position would mean for logosyllabic languages, such as Mandarin Chinese.

Another strong motivation for a reconsideration of the current framework in reading research comes from a few recent studies that have investigated in how far the putatively basic constituents of the reading process that have been identified in laboratory research actually apply to more naturalistic reading, that is, reading of connected texts. To that end, lexical variables such as word frequency during reading of connected text of several 100 or 1000 words were investigated. As we have discussed above, the word frequency effect is quite central in contemporary reading research, and states that words that occur more often in a language (i.e., possess a higher word frequency) are read faster. Word frequency is thought to capture a mapping between the visual appearance of a word and the associated memory strength for that word, again resting on the assumption of a word-level-semantics where the meaning of a word can be defined as a stable and elementary property in written language (Coltheart et al., 2001).

In two studies using different text and reader populations (Wallot et al., 2013; Wallot et al., accepted), it turned out that lexical variables such as word frequency explain only around a 0.001 to 1.0% of the observed variance in reading times (compared to 10–25% that are commonly observed in experimental studies on reading). So more is different, and taking two steps in a row might after all not be so similar to a days march – at least in the case of reading words and texts.

Other variables of principle theories of reading in psychology have not fared better when applied in the context of text reading (such as situation model variables that capture central aspects of sentence-level reading – McNerney et al., 2011). This strongly suggest, that if one wants to build a theory of reading, then perhaps other avenues have to be pursued – or as the authors of another study that investigated the transfer for psychological theories to connected text reading put it: “(…) we suggest, perhaps not surprisingly, that there is continued room for theoretical development to better capture the qualities of language that influence the ease with which it is understood” (McNerney et al., 2011).

Of course, the question is, where to look for new room? Maybe the right components of the reading process have simply not yet been found, and another lexical or sub-lexical feature will eventually solve the current problems. Alternatively, the very kind of stability that is expected of language and texts in reading research and the very basic assumptions of what reading really is might have to be reconsidered. In the next section, we want to pursue the second route, trying to evaluate the plausibility of some of the principle assumptions about reading that have been made in psychological reading research.

## DO WORDS HAVE MEANING?

As laid out before, contemporary psychology of reading views text as a decomposable communication, decomposable on the word level. That is, that stable word properties exist, and understanding of written text is first and foremost a decoding problem. This view of texts finds its complement in the component-processes in perception (e.g., word reading times, fixations, pronunciation times) and cognitive architecture (e.g., the mental lexicon) that mirror and match the supposed word-level structure of the text and vice versa (Wallot and Kelty-Stephen, 2014). As I noted above, the matching of a visual input of a word to its representation seems to satisfy the act of comprehending that word in contemporary reading research (a view that even seems to be shared by critics of the contemporary account – cf. Van Orden and Kloos, 2005). However, the lexical features that provide the informational basis for this mapping of visual input to representation are fairly static (within the human life-span), and for them to be of any value in this decoding process, the other end, that is the meaning of a word, needs to be similarly stable and static as well. The question is, whether such a view of meaning in written language is plausible – or asked differently: how can we say that words have meaning?

Before discussing the problem, a note is in order: clearly, if meaning is central to reading, then a definition of meaning is necessary in order to make headway toward a thorough theory of reading. My intuition is that a viable definition of meaning would need to go beyond language, and would need to have a wider basis in the interactions between organism and their environment (e.g., Turvey and Carello, 1981), also given that this has once been at the origin of reading and writing (Nissen, 1993). The problem of defining meaning is nothing that I will address in the current article, however, but it is also not necessary for the argument I want to make: what I want to examine is merely the option-space that one has to provide a workable definition of meaning (for written language), and whether this option space includes the possibility of defining meaning in terms of elemental features of words, or put differently: irrespectively of the current state of reading research, and even if one could simply wish for the findings that reading experiments would produce, would it make sense to define meaning as a stable property of words, which is required if one wants to explicate a theory of reading that is driven by objective features of written language.

As we have laid out, reading serves a communicative process and hence, reading ultimately needs to be about meaning. Furthermore, as I have tried to show, the psychology of reading conceives its basic constituents of a text and the perceptual and mental processes that act upon it mainly on the word level, and since these constituents need to be stable and context independent, this needs to be the level where meaning resides, encapsulated in words. There are several instantiations of how word meanings can be conceptualized in the different reading models, but the different versions are equivalent in that they seem to assume a locally definable meaning that will at best incrementally change in iterative learning processes^1^. The dominant ideas are that in a first step, there needs to be an associative process that relates visual word properties to some inner memory trace, such as provided by conceptual or neural networks. This way, the visual features that make up a word are connected to the word’s content (= meaning).

Several organizations of meaning of words have been proposed: either in the form of a mental lexicon, that possesses entries that are elemental meaning (i.e., the word “w1” has meaning “m1”) or definitions (i.e., the word “w1” possesses the definition “[w2, w3, w4]”). The definition can be a well-defined set of words (such as in strict views of the mental lexicon) or again some form of an associative network or matrix (where weights potentially connect one word to all other words, for example reflected in higher-dimensional theories of language such as HAL or LSA (Lund and Burgess, 1996; Landauer et al., 1998). Either way, the end product needs to yield a stable word content in order to lawfully connect the lexical word features to a word’s meaning.

Hence, the first question we need to ask is whether written language is decomposable into elemental meanings (on the word level)? The question of meaning has not been discussed in abundance among psychological researchers (Schvaneveldt, 2004). However, one can find occasionally a reference to Frege’s work, citing his axiom of composability (Bußmann, 1996) that states that the (literal) meaning of a sentence is composed of the meaning of its constituent words and their syntactical arrangement in the sentence. However, on a closer look, Frege’s thought on language is a little more complex than his axiom of composability suggests, for Frege complemented his axiom of composability with the axiom of context dependence, according to which a word cannot be defined or understood without knowing the sentence in which it is embedded in. At first glance, these two axioms seem contradictory. However, this contradiction can be resolved if one does not insist on the priority of the axiom of composability: if one knows the sentence in which words are embedded in, one can provide the meaning or definition for those words as they are used in the sentence. This seems an acceptable statement, and is one that could in principle be formalized, for example in impredicative logic (Aczel, 1988), and hence this resolution might even suit itself for a formal description of meaning in text^2^. The problem is, however, that this solution also does away with the word level as the basic level of the text and a basic level for the reading process – and with words as the carrier of a stable, well-definable and elemental meaning.

Another philosopher of language that was concerned with an elemental, stable level of meaning was Wittgenstein in his early work (Wittgenstein, 1922/1983). He proposed the idea of elemental sentences that form stable units of meaning (i.e., basic facts). However, as the term “elemental sentence” already conveys, the elements of meaning here are rather situated on the level of statements, incorporating relations between words in a sentence – not as a kind of elemental meanings in the sense of a word-token-semantics, but by virtue of a second step that relates the words to each other by virtue of their membership in that sentence. A word can only be considered as “elemental sentences” under certain circumstances, and, as Wittgenstein reasoned, the elemental sentences cannot be further reduced to their constituents (=words) and still be meaningful. This is due to the symbolic quality of words, that makes them arbitrary units (a view of language with which many reading researchers would agree). They are mere replacement characters, variables in a logical relationship, and can as such not be meaningful, because an arbitrary symbol does by definition not have a specific meaning. Accordingly, arbitrary symbols that stand in some relationship to each other will also not be meaningful.

This insight is a major problem for the conception of meaning in many reading theories: what all their conceptions of meaning have in common are not only that they are elemental, usually on the word level, but also that they are situated within a closed symbolic system: meaning is defined within a closed symbolic system either as an elemental relation (the meaning of word “w1” = “m1”), or as a composed definition (the meaning of word “w1” = “w2, w3, w4”). And as Wittgenstein also points out, a definition of a word by mere means of other words completes in the end a perfectly tautological cycle devoid of meaning. Substituting explicit definition by associations will also not solve the problem, for as Høffding already pointed out for the case of perception, associations alone cannot do any work, cannot create intelligence (Calkins, 1896) – or meaning. How to solve the problem?

## USING LANGUAGE

We left off with Wittgenstein’s description of the problem that meaning cannot be gotten within an encapsulated system of logical relations among symbolic constituents. How did Wittgenstein solve the problem? In his early work, Wittgenstein postulated a so-called picture theory of meaning (Wittgenstein, 1922/1983), stating that language is only in so far meaningful, as it refers to a fact, states-of-affairs in the world. With regards to the word-semantics view of meaning that seems to characterize contemporary reading research, the picture theory of meaning provides a twofold extension: first, it upscales the level of stability from the word-meaning-level to the level of statements. This is necessary, because in order to refer to states-of-affairs in the world, single words will usually not suffice, but a set of words and their relations to each other are necessary to provide sufficient reference. Second, it brings in meaning as a property that is co-determined by an environment outside of language, outside of a text, not within it. The interaction with the world is now a necessary precondition for meaning in language, a view that is also held by for example (Gibsonian) ecological psychology (Turvey and Carello, 1985). Still, there are also similarities: after all, Wittgenstein’s early thinking revolved around elemental sentences that describe states-of-affairs in the world as facts – basic facts, that are stable. And as soon as a proper reference (i.e., a proper sentence) has been constructed to specifically refer to a fact, it provides a meaningful building block. Hence, one could now search to operationalize elemental sentences (instead of elemental word properties) for a theory of reading. However, the idea of a basic meaning on the level of elemental sentences has received two blows. One with the general demise of logical positivism, more specifically the problems of verification (Popper, 2005), complicating the idea of a fact as a basic and stably describable property of the outside world. Another one with the development of Wittgenstein’s own thoughts about language in his late work.

In the Philosophical Investigations (Wittgenstein, 1953/2010), Wittgenstein expands and in parts revises his earlier positions. In further exploring the role of linguistic and non-linguistic context in language understanding, he arrives at a more complicated picture of meaning, that is not even stable on the level of elemental sentences, but is inherently dependent on context. Wittgenstein shows that there is a great number of contextual layers above the word or sentence level that need to be taken into account in order to know what a word or a sentence means (such as the larger set of utterances or paragraphs a sentence is embedded in, the shared individual and cultural history between interlocutors or readers, authors and texts, or the affordances of the actual and remote environment in which communication takes place). This now removes us very far from ideas of a general kind of meaning that could be encapsulated with small bits of written language, and moves us more into the realm of communication science and social interaction, that have picked up language use as a fundamental idea in communication. In a somewhat negative wording of the concept, one cannot get the meaning of a word or statement with knowing how it is used in a particular instance.

In communication research, the idea of language use refers to a great many different ways in which contextual constraints are effective or are utilized by interlocutors to arrive at a meaningful exchange. Some examples are the establishment of common ground, patterns of turn-taking during conversation, non-verbal clues and gestures, or linguistic alignment during conversation (Clark, 1996; Pickering and Garrod, 2004). These concepts are inherently relational and are not so much based on meaningful elements in language, but rather establish meaning by virtue of arranging and re-arranging the elements. Their relational quality is absolute in that if one removes from or exchanges one interlocutor or some relevant environmental feature in a conversation, they cannot be defined anymore in the same way or change their meaning.

Arriving this way at language use as a fundamental aspect of sense making casts up two problems for the current discussion of meaning: first, all these aspects of language use have been described for communication during online interaction, which is at the surface not quite how reading looks like – at least one needs to motivate an analogous way of conceiving the reading process. Second, among all the many aspects of language use in social interaction, how can we find a feature of language use that seems readily applicable to the reading situation and can be thought of as a fundamental aspect of language use in reading? Regarding the first question, i.e., how to motivate the analogy to language, we can ask whether there are certain similarities between the process of online communication (such as dialog) and the process of reading, that put the two in the same ball park.

Even though these proposals have largely been put forward with more interactive situations in mind, there seem to be some basic aspects that the two share: just as in a conversation where what is said and understood depends on the intentions of the interlocutors, reading depends on the intentions of the reader. This has consequences for how reading unfolds over time, behaviorally and emotionally, and what is remembered afterward from a text (Hunt and Vipond, 1991). Furthermore, reading depends on the assumed intentions of the author. The intentions of authors have also been carefully studied to make sense of written language across centuries of exegesis and are necessary in order to understand the meaning of ironic, satirical or metaphoric statements, which cannot be understood from their linguistic surface structure alone (Gibbs, 2002).

Similarly, it has been pointed out that just as “The possibility of language, thought, and interpretation depends on the triangular situation which relates speaker and listener, and both to a shared object in the public world which they can observe together, and to which they can observe each other’s responses. Such a triangular situation exists in literature. Interpretations of a text will vary from person to person, culture to culture, and century to century. However, it does not follow that a text means whatever its readers take it to mean, since disagreements about the meaning of a text are only possible against a shared basis of agreement” (Davidson, 1993), highlighting how the process of reading as a communicative process is related to other forms of communication, such as conversation, and how intersubjective contexts necessarily factor into the reading situation.

Turning back to contemporary reading research, these assertions bring up the interesting question of how one should judge the experimental situations that are dominantly used to study reading from this perspective? As most reading tasks feature reading of isolated, random letter strings, or a few sentences at the most, many of the aspects that are considered necessary pre-condition for the reading process in literary studies and the philosophy of languages are virtually absent in the empirical investigations of reading.

These doubts set aside, there are not only interesting conceptual commonalities between reading and conversation as communicative processes, but they also share similarities with respect to their dynamic structure, with respect to basic patterns of behavior that can be observed in both: both exhibit kinds of feedback loops in behavior, such as in conversations where interlocutors go back and forth to clarify terminology and topic, until common ground is established (Clark, 1996). In reading, similar feed-back loops are evident to secure proper understanding of a text, such as re-reading previous text passages (Rayner et al., 2006), together with reflective thought processes which now substitute for communicative exchange. Similarly, when understanding is jeopardized or a new topic is introduced, one observes disruptions of otherwise rather “smoothly” proceeding processes in conversation as well as in reading. As we will see in the next two sections, such dynamic aspects of the reading process will be important to provide a measure of basic aspects of language use that can be employed in reading research. But first we will need to find a core concept of language use that motivates a particular operational definition that can be employed in reading research, and that provides an interpretational dimension for its measures. In the next section, I will briefly introduce Wittgenstein’s concept of “language games,” and show how it captures a basic aspect of language use that can be applied in the context of reading.

## LANGUAGE GAMES: A FUNDAMENTAL ASPECT OF LANGUAGE USE

Wittgenstein introduces “language games” in his Philosophical Investigations as a concept that holds together the great diversity of language activities that can be observed. After describing the sensitivity of meaning in language to the various contextual constraints that one can identify, Wittgenstein reasoned that natural language-use is not governed by a general process that underlies all of those instances, but that it is inherently dependent on historical, social and other contextual factors (Wittgenstein, 1953/2010). Hence, natural language is not a homogenous category that can be defined by a small set of general language-rules or language-elements that hold across all contexts, but it is rather composed of different classes of language use, for which Wittgenstein coined the term “language game.” In analogy to real games, Wittgenstein pointed out that language games possess rules according to which language is used within each game, but that different games differ in terms of the rules that govern use. Furthermore, the rules observed in each game are emergent. That is, even though they seem to have real causal power within the same family of language games, they do not point to any fundamental principles of how language generally works. The rules do not transcend the boundaries of the particular language game within which they are observed.

Again, when reading the Philosophical Investigations, it seems clear that Wittgenstein had more social, dialogical situations in mind, than a person reading a book. However, as we have briefly discussed in the previous section, reading, and conversation share a good deal of conceptual and behavioral similarity, to warrant an analogy between language games and “reading games.” If we try to use the idea of language games for reading, then we are interested in how this concept relates to the current state of reading research and its challenges. Furthermore, of course, we are also interested in how it can be utilized for the empirical investigation of reading.

Regarding the question of how reading games tie in with the state-of-affairs in reading research that was summarized at the beginning of this manuscript, the crucial point to take from the application of language games to reading is that the differences between two reading games (that is, reading situations such as reading silently or aloud, reading in English or reading in Hebrew, or reading prose or poetry), can be both, quantitative and qualitative: reading games that belong to the same family exhibit similar rules, while reading games that belong to different families abide by potentially completely different sets of rules (Wittgenstein, 1953/2010).This makes immediately understandable why the effect-landscape observed in current reading research is so heterogeneous: contextual variations and experimental manipulations do sometimes not just constitute mere quantitative changes in the manipulated factor, but can effectively constitute a change from one type of reading game into another, changing reading qualitatively. However, in the absence of a definition of the boundary conditions within which a particular reading game is stable and only quantitative variations occur, a particular experimental variation that looks rather moderate from the perspective of the researcher (such as reading a word silently or aloud – Forster and Chambers, 1973) can tip a reading game, not just changing a particular aspect of that game, but turn it into a new game that works according to entirely different rules altogether. If one buys into reading games as a fundamental concept, this explains why reading research is so diverse, and scientists keep being surprised by entirely unexpected context effects, for instance the relative insensitivity of the reading process to letter-position in some languages (Frost, 2012) or the continued interaction between task-aspects and reading performance that stress or suppress the role of phonology in reading (Van Orden and Kloos, 2005).

Also, I have stressed the tension between single-word and connected text reading. However, there are of course instances were naturalistic reading is reading of only one word, and the concept of reading games gives a proper role to the case of single-word reading in naturalistic settings: even though the case of text-reading seems to be the standard that reading research aims at explaining, everyday life is of course full of examples where only one or two words convey information. For example imagine somebody sitting in a restaurant and starting to feel the sudden urge to visit the restroom. Of course, this person will get up, look for a sign saying “restroom” (or “toilet,” or “WC”), and have that sign guide their searching behavior. The sign means then “toilet,” or perhaps “place where on can relieve oneself in private.” However, the understanding of the word is a function seminar to the triadic relation that Davidson (1993) described, where the reader needs to have a certain intention with regard to the word “restroom,” the word needs to be presented in a particular context, and the intention of the (proximate) author, in our case perhaps the restaurant owner, to provide guidance for her customers. Imagine the person seeking for a restroom sees the word “restroom” as part of an advertisement for American Standard water closets. This will surely evoke a different behavior and understanding of that word.

Furthermore, one must not forget that investigating how a person understands a word for scientific psychology means observe behavior in response to the word, and different contexts (“restroom” in the context of restroom and “restroom” in the context of advertisement) will elicit very different behaviors. While this is intuitive for understanding in these everyday situations, one has to wonder what participants in psychological laboratory tasks understand when they read random word lists on a computer screen – what are the intentions of the participants with regard to/regarding the text stimuli, and what are the intentions of the author that factor into reading here?

Regarding the question of how the concept of reading games can be used for the empirical investigations of reading, the crucial point to take away is that what is unifying across reading games is not the presence of a particular set of rules that applies throughout all contexts (such as that high frequent words that occur often in a language are read faster compared to low frequent words), but that reading games are always rule-abiding, exerting a structuring effect on reading behavior (such a set of locomotion patterns for somebody looking for a restroom in response to the sign “restroom”). This rule-abiding aspect of reading games can serve as a new fundament for reading research and solve the outlined problems – i.e., what is the common core across systematic idiosyncrasies in reading behavior between different readers, what is common across reading in different languages and situations, and how one can define the (text-)reading process in the absence of a strong and stable relationship between surface properties of the text (such as lexical word features) and reading behavior.

Regarding reader idiosyncratic differences in reading behavior between readers, the reading game conception would allow us relax the degree of detail that we need to explicitly address, for example when investigating the question whether two readers that read the same text but in very different ways (i.e., many short saccades and fixations with frequent regression compared to few long fixations and saccades with few regressive eye-movements) possess a similar or different degree of aptitude in reading or comprehension of a text. From the reading game perspective, we could hypothesize that the better a reader abides by the rules of a reading game, the better she is able to utilize (con-)textual information and thus the better of a reader she is. That is, no matter what form the specific rules of a reading game take, the more one abides by the rules, the better the game is played – no matter what specific behavioral pattern a reader exhibits during reading, the key-question is to what extent this patter reflects a rule-abiding reading process or not.

We can make a similar argument for the other two cases, reading in different languages and text reading: reading in two languages might exhibit differences in how readers utilize certain word features (e.g., English vs. Hebrew), and some writing systems might even exhibit word features that nothing others do not even possess (e.g., English vs. Mandarin), but proficient reading should always be a structured, rule-abiding activity, as there will be some systematic aspects in the relation between reader and text within each writing system. Similarly, for text reading, we would require that a reader who reads and understands a text exhibits some form of structured behavior during reading, even if this structure cannot be pinned down to specific features of the text in a general manner.

Furthermore, the concept of rule-abidingness in reading games might be used to empirically sort-out the boundary conditions up to which the same reading rules apply (e.g., that high frequent words are read faster), or at which they change (e.g., that high-frequent words are read substantially faster in isolated word reading tasks, but not during connected text reading – Wallot et al., 2013). This can be done because reading games provide us with a bottom-up definition of the boundary conditions between any two qualitatively different reading contexts: when a reader moves from one reading game to another that differs in rules, then this will lead to a disruption of rule-abiding behavior at the transition-point between the two games, as the established rules of the first game are broken while the new rules of the second game are still being established. In contrast, if a reader moves between two reading games that share the same set of rules, the degree of rule-abidingness will remain stable at the transition-point between games. However, in order to test such a hypothesis, one needs an operational definition of a reading game to measure such effects in empirical data. In the next and last section of this manuscript, I consider possible statistical operationalizations of the reading game concept, and review some preliminary evidence of the utility of the concept.

## READING GAMES: POSSIBLE OPERATIONALIZATIONS

Following the conceptual clarification, what is needed is an operationalization of the concept of reading games, or to be more precise, the degree of rule-abidingness in measures of reading. Here, an important note is in order: when thinking of rule-abiding reading behavior, it is not implied that the rules are consciously understood or explicitly followed by the reader. What is rather meant, is that measures of reading behavior in a particular context are not random, but follow systematic patterns that can be formulated as a rule by an observer, such as “the higher the frequency of a word, the faster that word is read by a reader.”

The conception of rule-abidingness in a reading game that is presented here is in some respect very similar to the standard assumptions that go into current theories of reading: if a reader aptly reads a text (or word), comprehends it (sufficiently) and acts in accordance with it (for example by moving their gaze further along a text, or opening the door that leads to a restroom as opposed to out into the kitchen), this implies that the text constraints the reader’s (reading) behavior, and that the reading behavior that can be measured is somehow coupled to the text. However, if the reading game analogy holds, then it will – for many cases – be problematic or impossible to formulate the other side of the equation in a general manner, that is, to what aspects of the text the reader’s behavior is coupled to and in what way.

As I have discussed, simple reading tasks that allow next to zero variation on the side of the reader and carefully try to investigate only one factor of reading at a time already fail to yield a stable pattern of general mechanisms that guide the reading process. One should not expect to fare any better as the complexity of the stimulus material and the degrees of freedom on the side of the reader are scaled up. Hence, quantifying the degree of order in reading behavior is an attempt to define the degree of coupling between text and reader when access to only one of the two is possible.

Hence, we seek a measure of rule-abidingness that tells us how structured a particular measure of reading (e.g., eye-movements or prosody of a voice record during reading) is without having to specify where the structure comes from in detail. Such measures can be taken from the toolbox of statistical physics (such as permutation entropy analysis, Bandt and Pompe, 2002; cross-convergent mapping, Sugihara et al., 2012; recurrence quantification analysis, Webber and Zbilut, 2005; or fractal characteristics, Wallot et al., 2012). These methods provide measures of the degree of temporal structure and predictability in time-series, and could lend themselves to an operational definition of rule-abidingness of language games, because they extract and quantify the degree of temporal structure without the need to define the rules *a priori*. Furthermore, they are non-linear methods that are able to detect rules in time-series that do not follow any simple, obvious patterns, such as chaotic time-series that appear random, but are in fact deterministic (Zbilut et al., 1998). This is important because the crux with strong unexpected context effects is that when they occur, the rules of the new context are not yet well understood, and can thus not be easily formulated on the grounds of the rules observed in previous contexts. However, as has been described above, by simply quantifying the degree of temporal structure, we are able to distinguish between different reading games, also in the absence of more detailed knowledge.

**FIGURE 1:**
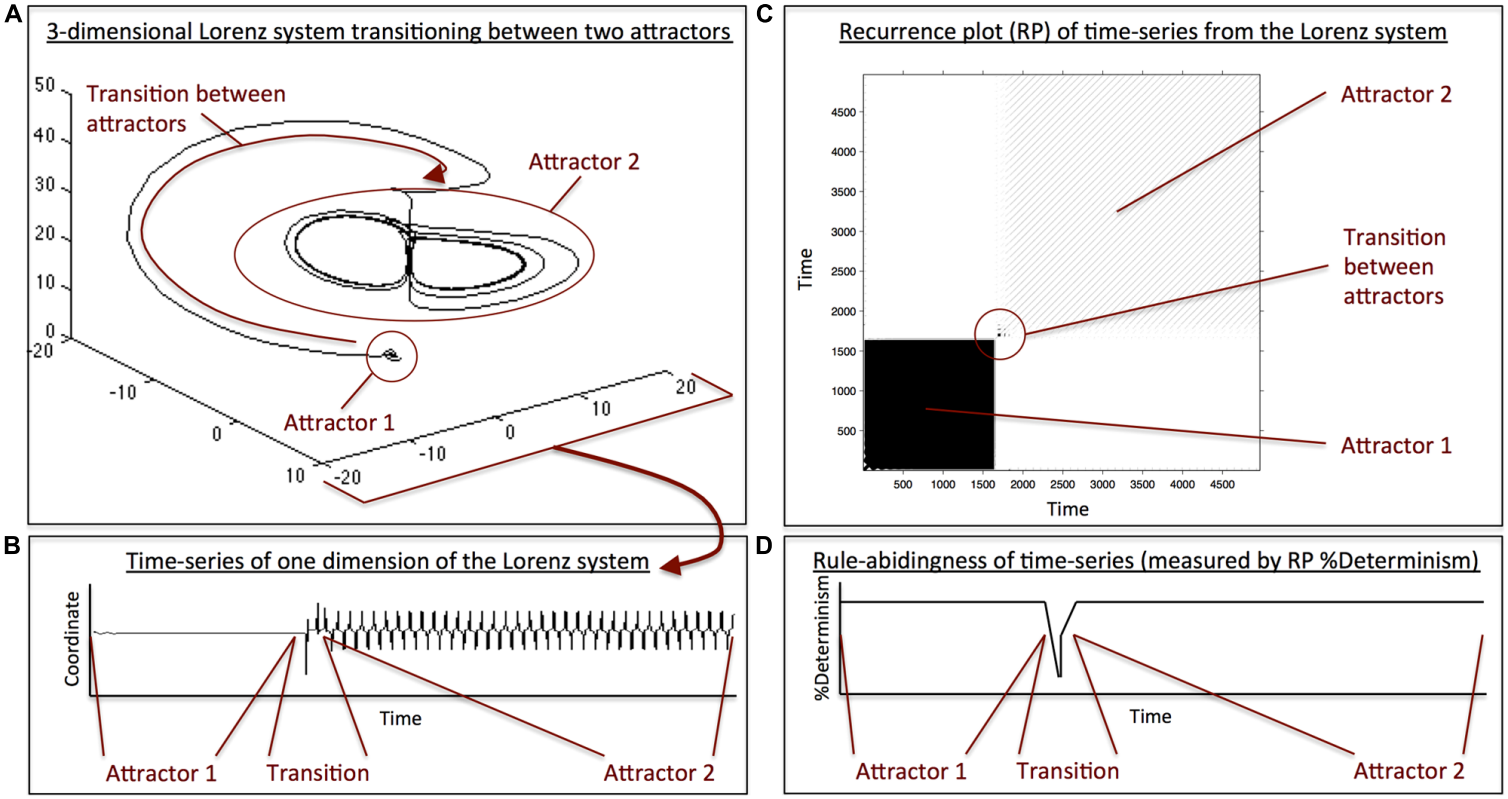
**(A)** 3D illustration of a switching between two attractors (i.e., two quantitatively different types of behavior) in the Lorenz system, a coupled differential equation system consisting of three equations. When going through a phase-transition (i.e., moving from one attractor to another), the system does not show a smooth or instantaneous transition between the two attractor-states, but produces a transition period with major displacement. **(B)** Time-series of a single dimension of the Lorenz system that shows the behavior in the first attractor, the behavior in second attractor, and the transition phase. The behavior within each attractor looks very different, and the transition phase is marked by a period of increased fluctuation. **(C)** Recurrence plot (RP) of the time-series in **(B)**. Recurrence plots are 2-dimensional representations of a time-series where time moves from the lower-left part of the plot to the upper-right part along the diagonal of the matrix. Dark areas in the plot indicate a high degree of temporal structure in the behavior of the time-series. White areas represent the absence of temporal structure in the time-series. The RP is similar to an autocorrelation plot, where time at lag0 runs along the diagonal. As one moves away from the diagonal toward the upper-left or lower-right part of the plot, one sees time-lagged behavior. Hence, the plot shows that the initial behavior (i.e., behavior in attractor 1) is highly structured, indicated by the dark area in the lower-left. Similarly, behavior in attractor 2 is highly structured, indicated by the striped area in the upper-right. However, the transition period between the two attractors is marked by a brief absence of structure. **(D)** Illustration of an RP-based measure of structuredness (%Determinism) of the time-series in **(B)**. For both attractors, 1 and 2, the time-series possesses a high degree of temporal structure, but the transition between both attractors is marked by a loss of structure, indicated by the dip in %Determinism.

The example in **Figure 1** illustrates this point by showing how such a measure of temporal structure can be used to capture transitions between two qualitatively different behaviors. The data was generated by the Lorenz system, an equation system that consists of three coupled differential equations (**Figure 1A**). Depending on the parameterization of the system, it is capable of exhibiting different types of dynamics. When the parameters are changed accordingly, the system transitions from one type of behavior (a stable fix point) to another (oscillating behavior). This is also evident in a one-dimensional “measurement” of the system (**Figure 1B**), which could be thought of as analogous to a time-series of word reading time during text reading, for example. To quantify the degree of temporal structure, the one-dimensional time-series can be represented as a recurrence plot that shows the degree of structure within that time-series (**Figure 1C**). From the recurrence plot, one can now derive statistics of temporal structure (Webber and Zbilut, 2005) in the original time-series (**Figure 1D**). As can be seen, both types of behavior exhibit a high degree of temporal structure, but the transition point between them is marked by a brief loss in that structure, indicating a change from one behavior to the other. This example does not only illustrate how a change in temporal structure can be detected, it also highlights another important point about the reading game concept: as mentioned earlier, Wittgenstein described that the rules observed in language-use dot not point to the foundations of language, but are themselves emergent features of language-use-in-context. Similarly, the Lorenz system can exhibit oscillatory behavior, but the fact that oscillations are observed is not in a straight-forward way informative about its architecture, as it, for example, does not include a sinusoidal function. When the behavioral rules are emergent, such as the oscillations in the Lorenz system, and the system moves from one type of behavior to another, then the transition is unsmooth, creating an abrupt drop in the structuredness of behavior (Kelso, 1995). Hence, if the rules that govern reading behavior are similarly emergent, as the concept of reading games holds, then such transitions will necessarily occur when shifting between two qualitatively different reading contexts, predicting the formation of a new reading game.

**FIGURE 2:**
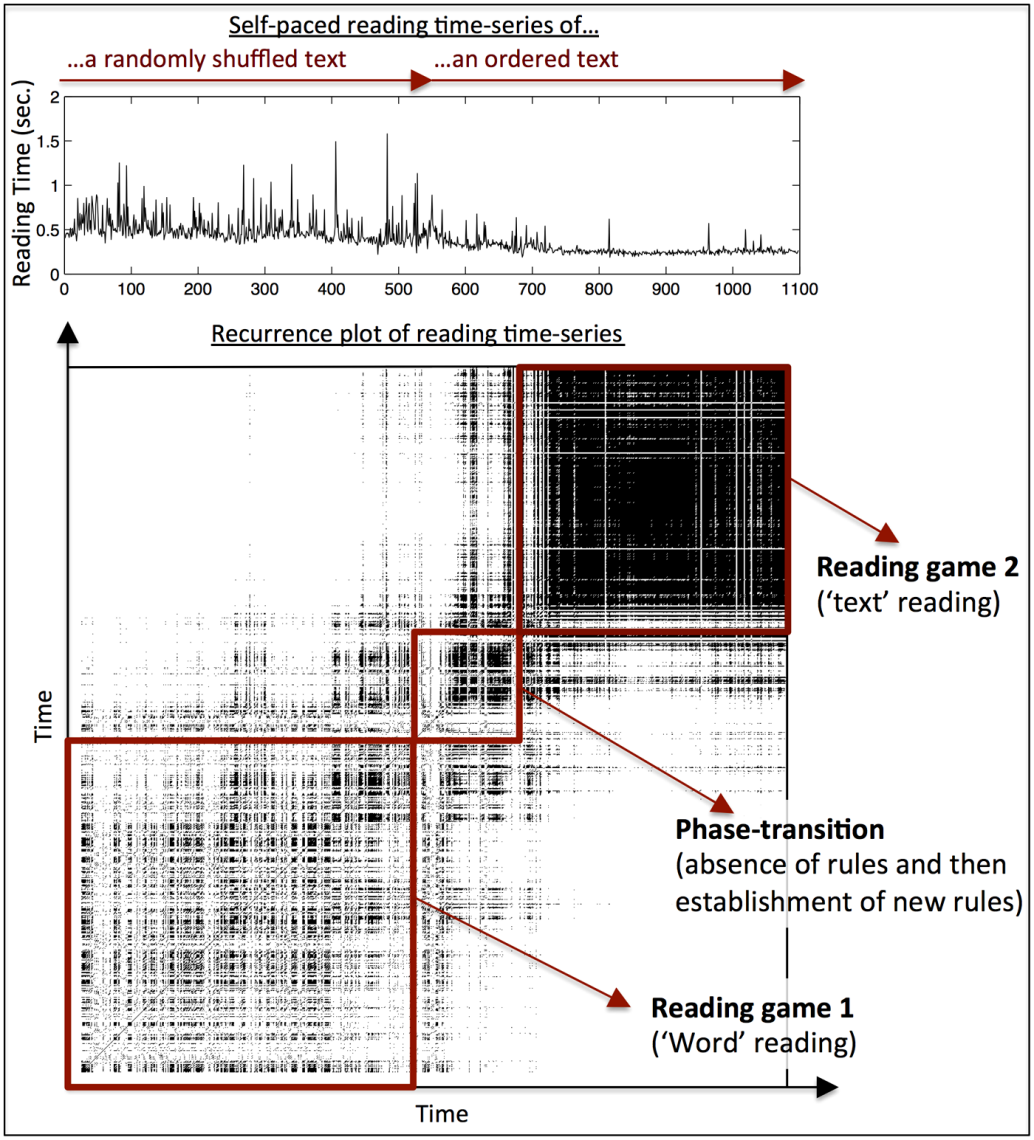
**Pilot data from one participant during self-paced reading.** The first 530 words were presented as randomized words. Thereafter, words appear as an ordered connected text. The upper panel shows the time-series of word reading times. The lower panel shows the RP of that reading time-series. Similar to the change between attractor-states presented in **Figure 1**, one can see that reading of randomized words (reading game 1) and reading of a connected text (reading game 2) are both temporally structured (as evident by black areas in the plot). Furthermore, the transition from random word reading to connected text reading is neither instantaneous, nor smooth, as indicated by the transition-phase that connects the two reading conditions. Lastly, it can be seen that random word reading and text reading are different in terms of how they are temporally organized, as the off- diagonal areas in the upper-left and lower right area are white, indicating no shared temporal structure between word and text reading.

To illustrate the effect for reading, I collected a set of pilot data to provide a proof-of-concept of the reading game proposal, namely that the degree of temporal structure in reading can provide a bottom-up definition of the boundaries between two reading games. In a self-paced reading task, participants read a text of 1099 words. The first half of the text was randomized, effectively providing an individual word reading task that is used in most reading studies, while the second half of the text was left intact. The time-series of reading times is displayed in a recurrence plot (as in **Figure 1**), which is used to compute temporal structure within a reading time-series. As can be seen in **Figure 2**, individual word reading and text reading appear as two qualitatively different tasks: while each of them shows a specific global reading pattern, there is basically no overlap between those patterns, as indicated by the white spaces to the upper-left and lower-right off the main diagonal. Furthermore, this distinction also predicts a “change in rules” between the two tasks: while word frequency plays a substantial role in individual word reading (*R*^2^ = 0.046; *p* < 0.001), this decreases to a marginal effect in text reading (*R*^2^ = 0.006; *p* = 0.061).

Furthermore, in a recent set of studies, we utilized recurrence quantification analysis on text reading data to assess reading performance of children and adults and for the prediction of text comprehension: in one study (O’Brien et al., 2014), children (2nd, 4th, and 6th graders) and adults read a simple children’s story silently or aloud in a self-paced manner. That is, participants always pressed a button to reveal each new word of the story, read the word, and pressed the button again to reveal the next word of the text. Hence, the intervals between two consecutive button presses estimated the reading time of that word (Just et al., 1982). It was found that recurrence measures that quantify the temporal structure in reading times increase with age and distinguish better between readers of different age than reading rate. In an investigation of reading process predictors of text comprehension (Wallot et al., accepted), we found that the degree of temporal structure of reading times turned out to be a good predictor of text comprehension in both, silent and oral reading, and again better than reading speed. A third study on the effect of repeated reading also found that repeated text reading, which is thought to increase reading fluency for that text, led to increases in temporal structure of reading times for less skilled readers, even though the pattern of effects was not as clear as in O’Brien et al. (2014) or Wallot et al. (accepted). Generally, these results fit with the reading game conception, where rule-abidingness – as measured by the degree of temporal structure in reading times – indicates mastery of a reading game and thus should relate positively to reading skill and text comprehension. Moreover, these findings tie in with new research on conversation during dyadic interaction, where the degree of temporal shared structure in utterances between interlocutors positively correlated with the success of the interaction on a shared decision making task (Fusaroli and Tylén, under review). This seems to indicate that temporal structure lends itself as a measure of rule-abidingness, which can serve as a general indicator of skilled language use – be it reading or conversation – in the language game conception.

Of course, the evidence presented comes from a single case of reading or is based on retrospective interpretations of already published work, and proper prospective data to test some of the basic predictions of the language game conceptions need to be collected. Nevertheless, these findings lend some motivation that the concept of reading games can serve as a fruitful and fundamental property of reading, that circumvents some of the conceptual problems of contemporary theories of reading, especially their take on meaning. To utilize and explore the value of this conception, first investigations are needed that solve basic measurement issues, such as what measures of temporal structure (e.g., RQA, CCM, entropy measures, correlation dimensions) make for sensitive and reliable operationalizations of rule-abidingness, and whether and how they converge. Then, subsequent investigations might shed light at more specific hypotheses, such as whether rule-abidingness can be used to predict differences between qualitatively different reading tasks, or serve as a general metric for skilled language use across readers, texts and languages, and connect reading to back to the broader field of human communication.

## Conflict of Interest Statement

The author declares that the research was conducted in the absence of any commercial or financial relationships that could be construed as a potential conflict of interest.

## Footnotes

1 Just as a side note, one has to say that it is not the case that psychological investigations did not also find evidence that word meaning is more flexible than that – or of changes within a single “learning instance” or less – however, these findings have persistently remained outside of the scope of the dominant and well-developed theories of reading in psychology.

2 For the sake of clarity, it should be noted here that such a kind of formalization would not lead to a simple primacy of one axiom over another, or the sentence level over the word level, but would rather yield a mutual relationship between the two, but a relationship where one level cannot be reduced to the other.
